# Present trends, research gaps, and emerging priorities in falcon studies: A four-decade bibliometric analysis from the Arabian Gulf

**DOI:** 10.14202/vetworld.2025.1452-1465

**Published:** 2025-06-06

**Authors:** Ahmed Alsaleem, Mahmoud Kandeel, Mohammed Al-Rasheed

**Affiliations:** 1 Department of Biomedical Sciences, College of Veterinary Medicine, King Faisal University, Al-Ahsa, Saudi Arabia; 2 Department of Clinical Sciences, College of Veterinary Medicine, King Faisal University, Al-Ahsa, Saudi Arabia

**Keywords:** Arabian Gulf, bibliometric analysis, conservation genetics, falconry, molecular epidemiology, research mapping, veterinary science

## Abstract

**Background and Aim:**

Despite the profound cultural and ecological significance of falcons in the Arabian Gulf, systematic evaluations of regional falcon research are lacking. A bibliometric approach can elucidate the evolution, influence, and emerging priorities within this specialized field. This study aimed to provide the first comprehensive bibliometric mapping of falcon research in the Arabian Gulf countries over the past four decades (1984–2024), revealing research dynamics, international collaboration networks, thematic trajectories, and critical knowledge gaps.

**Materials and Methods:**

Original research articles were systematically retrieved from the Scopus database using targeted search strategies restricted to title-level keywords and affiliation filters. Following Preferred Reporting Items for Systematic Reviews and Meta-Analyses guidelines, 126 articles were selected for analysis. Bibliometric methods – including co-authorship networks, keyword co-occurrence, thematic mapping, and trend analysis – were applied using VOSviewer, Bibliometrix, and Excel.

**Results:**

The research output exhibited a modest annual growth rate of 2.32%, predominantly driven by contributions from the United Arab Emirates and Saudi Arabia. Strong international collaborations (56.35% co-authored publications) were identified, particularly with European and North American institutions. Research themes historically centered on clinical veterinary topics and avian biology are now shifting toward molecular diagnostics, genetic studies, and disease surveillance. However, a clear regional imbalance and underrepresentation of emerging fields such as genomics, epidemiology, and conservation breeding were observed.

**Conclusion:**

This analysis underscores the need for an integrative, regionally inclusive research strategy incorporating advanced molecular technologies and conservation science. Strengthening cross-border collaboration, adopting genome-based monitoring, and addressing emerging infectious threats will be critical to advancing falcon research and preservation in the Arabian Gulf.

## INTRODUCTION

Falcons and the practice of falconry hold a distinguished place in the cultural and historical heritage of the Arabian Gulf countries [1]. This enduring relationship is reflected across multiple facets of cultural expression [2], including literature, poetry, art, and national symbols. Falconry, recognized by the United Nations Educational, Scientific and Cultural Organization as an Intangible Cultural Heritage [3, 4], continues to thrive in countries such as Saudi Arabia, the United Arab Emirates (UAE), Qatar, and Oman, where it is actively celebrated through festivals, compe-titions, and dedicated breeding programs. Beyond their cultural significance, falcons in this region possess considerable ecological importance. The Arabian Gulf’s unique environment – characterized by expansive deserts and mountainous terrains – provides critical habitats for a variety of falcon species. In response to habitat degradation, illegal hunting, and urbanization, conservation initiatives have intensified across the Gulf countries in recent years [2, 5, 6].

Research on falcons within the Arabian Gulf region has primarily concentrated on their health, behavior, and conservation, encompassing both local and broader geographic contexts. A substantial body of work has addressed various diseases and health conditions affecting falcons, including fungal infections [7] and parasitic infestations [8]. These studies encompass diagnostic methodologies [9], therapeutic interventions [10], and vaccine development [11], underscoring ongoing efforts to preserve and enhance falcon health, particularly within falcon populations in Saudi Arabia, the UAE, and Oman [5, 7, 12, 13]. In addition to health-focused research, significant attention has been directed toward the genetic and ecological study of falcons, encompassing investigations into breeding behavior [14], migratory patterns [15], and population dynamics [16]. These efforts have incorporated advanced methodologies such as satellite telemetry for migration tracking [15], genetic analyses to elucidate genome structure, evolutionary history, and species relatedness [17, 18], and studies examining the environmental factors influencing falcon reproduction [19].

The conservation dimension remains a promi-nent theme, with numerous studies emphasizing the role of captive breeding programs in species conse-rvation, the management of endangered falcon populations, and the broader implications for regional biodiversity [5, 6, 19, 20]. Given the expanding scope and complexity of falcon research, bibliometric analysis has emerged as a critical tool for systematically assessing the dynamics, trends, and impact of scientific inquiry within this specialized field [21–24]. Such analyses offer valuable insights into research trajectories, collaboration networks, and the influence of scholarly publications, thereby informing strategic directions for future research and conservation efforts.

Despite the rich cultural heritage and ecological importance of falcons in the Arabian Gulf, scientific research on falcons in the region remains fragmented and limited in scope. Existing studies have largely concentrated on clinical aspects, such as infectious diseases and therapeutic management, as well as basic ecological investigations. However, comprehensive evaluations of research productivity, thematic evolution, collaboration patterns, and emerging scientific priorities are notably lacking. Furthermore, critical areas such as genetic diversity, molecular epidemiology, captive breeding strategies, and conservation genomics rem-ain underexplored, particularly when compared to the increasing global interest in avian health and biodiversity preservation. The absence of a systematic bibliometric analysis impedes a clear understanding of the research landscape, hinders the identification of knowledge gaps, and constrains strategic planning for future multidisciplinary research efforts across the Arabian Gulf countries.

This study aims to systematically map and critically evaluate the research landscape of falcon studies conducted in the Arabian Gulf countries over the past four decades (1984–2024) using advanced bibliometric techniques. Specifically, it seeks to analyze publication trends, assess author and institutional collaborations, identify core research themes and their temporal evolution, and highlight emerging and declining topics within the field. By providing a comprehensive overview of scientific productivity, collaboration networks, thematic developments, and research frontiers, this study intends to inform future research strategies, foster regional and international collaboration, and support evidence-based conservation and health initiatives targeting falcon populations in the Arabian Gulf.

## MATERIALS AND METHODS

### Ethical approval

Ethical approval was not required for this study, as the research involved the analysis of previously published data sourced from Scopus and did not involve human participants, animal subjects, or unpublished confidential information.

### Study period and location

The study was conducted in September 2024 in Alahsa, Saudi Arabia.

### Registration

This study was prospectively registered with the Open Science Framework (OSF) under the title “Mapping Four Decades of Falcon Research in the Arabian Gulf: A Bibliometric Analysis.” The registration record is publicly accessible at https://osf.io/8kz3t/ and includes a detailed protocol outlining the study’s objectives, methods, inclusion and exclusion criteria, and planned analyses. No modifications to the pro-tocol were made after the commencement of data collection.

### PICO framework

#### Population (P)

Original research articles focused on falcons (Falco spp.) conducted in the Arabian Gulf countries (Bahrain, Kuwait, Qatar, UAE, Saudi Arabia, and Oman), published between 1984 and 2024.

#### Intervention (I)

Systematic bibliometric analysis assessing resea-rch productivity, collaboration patterns, thematic evol-ution, and emerging trends using data retrieved from the Scopus database.

#### Comparison (C)

It is not directly comparative; however, it involves internal comparisons across countries (e.g., UAE versus Saudi Arabia) and over time (early years versus recent years) to evaluate growth, collaboration intensity, and thematic shifts.

#### Outcome (O)

Mapping of research output trends, identification of leading authors and institutions, visualization of collaboration networks, thematic classification of research areas, and recognition of emerging research priorities and knowledge gaps.

### Data collection and dataset characteristics

**Figure 1:**
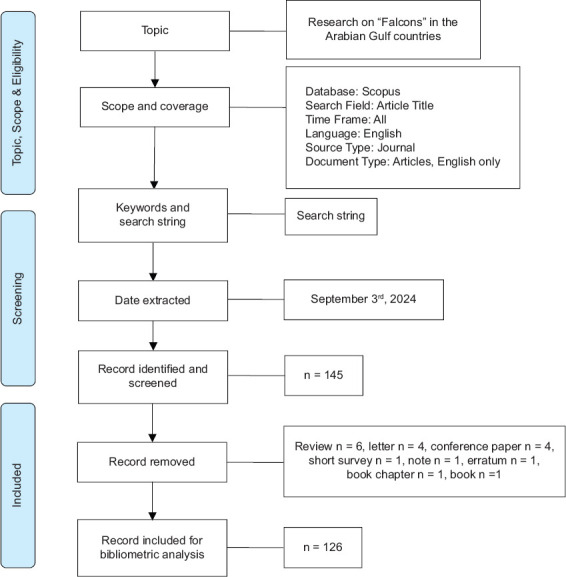
Preferred Reporting Items for Systematic Reviews and Meta-Analyses flow diagram for the selection of Falcon research studies in the Arabian Gulf Countries.

A systematic methodology, illustrated by the Preferred Reporting Items for Systematic Reviews and Meta-Analyses flow diagram (Figure 1), was employed to identify, screen, and select relevant studies for a bibliometric analysis of falcon research in the Arabian Gulf countries. The initial search was conducted in the Scopus database using specific keywords and search strings. The search strategy applied was: TITLE (falcon) AND (LIMIT-TO (AFFILCOUNTRY, “Bahrain”) OR LIMIT-TO (AFFILCOUNTRY, “Kuwait”) OR LIMIT-TO (AFFILCOUNTRY, “Qatar”) OR LIMIT-TO (AFFILCOUNTRY, “United Arab Emirates”) OR LIMIT-TO (AFFILCOUNTRY, “Saudi Arabia”) OR LIMIT-TO (AFFILCOUNTRY, “Oman”) AND LIMIT-TO (DOCTYPE, “ar”). This search retrieved a total of 145 records.

During the screening phase, all identified records were critically reviewed to ensure compliance with pre-defined eligibility criteria. Nineteen records were excluded at this stage, including reviews, letters, confer-ence papers, short surveys, notes, errata, book chapters, and books. The exclusion of these publication types was intended to maintain a focus on original research articles that directly contribute to the understanding of falcon-related topics within the Arabian Gulf region. The final dataset comprised 126 original research articles, which formed the basis for subsequent bibliometric analyses.

### Inclusion and exclusion criteria

The inclusion criteria for the bibliometric analysis were as follows:

1. Only original research articles focused specifically on falcons within the Arabian Gulf countries were included.
2. Articles were required to be published in English.
3. Only articles indexed in the Scopus database were considered.
4. The search was restricted to articles with relevant keywords appearing in the title field.

Conversely, the exclusion criteria eliminated studies that did not align with these specifications. Reviews, letters, conference papers, short surveys, notes, errata, book chapters, and books were excluded to ensure that the analysis was based solely on primary research outputs. In addition, studies that were not geographically centered on the Arabian Gulf region or authored by researchers outside this context were removed, along with non-English publications, to mitigate potential language bias.

### Bibliometric techniques

The bibliometric analysis followed a structured and systematic approach involving several key techniques. Following data collection, the dataset was subjected to quantitative and network-based analyses to explore research trends, collaboration patterns, and thematic developments within the field. Keyword analysis was performed to identify the most frequently occurring terms and major research themes. Co-authorship analysis examined collaborative relationships among individual researchers and institutions, while journal analysis evaluated the impact and influence of different publication sources. Thematic mapping and trend analysis were also conducted to visualize the progression of research topics over time and to detect emerging areas of interest.

### Tools and software

The collected bibliometric data were processed and analyzed using VOSviewer (version 1.6.20, https://www.vosviewer.com/) and the Bibliometrix R package (R version 4.4.1, https://www.r-project.org/). VOSviewer was utilized to construct and visualize networks for co-authorship, citation relationships, and keyword co-occurrence. Bibliometrix facilitated thematic mapping, trend analysis, and detailed citation analysis. In addition, Microsoft Excel (Office 365, Microsoft Corporation, USA) was employed for data cleaning, dataset organization, and generation of descriptive statistics to support the visualization and interpretation of bibliometric outcomes.

## RESULTS

### Characteristics of falcon research in the Arabian Gulf countries

**Figure 2:**
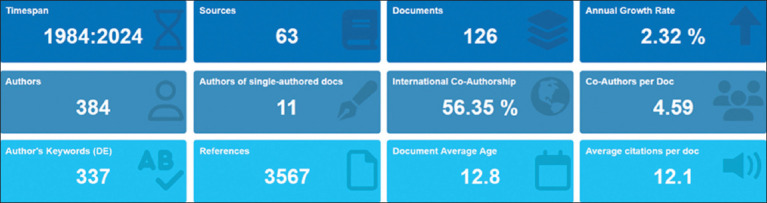
The data set characteristics of research on falcons in the Arabian Gulf countries.

The dataset characteristics of falcon research in the Arabian Gulf from 1984 to 2024 reveal important insights (Figure 2). Over the 40-year period, 126 documents were produced by 384 authors, with an average of 4.59 co-authors per document, indicating substantial collaborative efforts, with 56.35% of publications involving international co-authorship. Research outputs were disseminated across 63 different sources, with an observed annual growth rate of 2.32%. This growth rate reflects a relatively modest expansion compared to other veterinary bibliometric studies, where higher rates were reported for camel research in North Africa (7.18%) [25] and China (8.05%) [26]. Despite the relatively low number of single-authored papers (n = 11), the field demonstrates notable impact, with an average of 12.1 citations per document and a cumulative reference count of 3,567. The documents had an average age of 12.8 years, suggesting a mature but continually evolving research field.

### Research timeline

**Figure 3:**
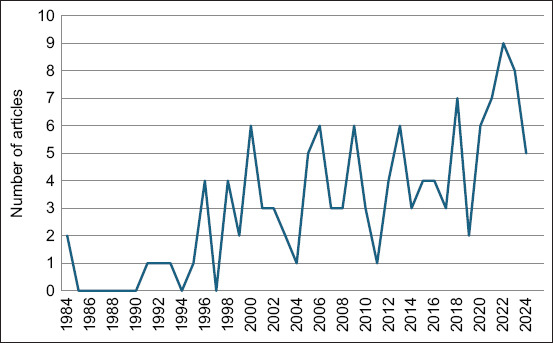
Annual scientific production of research on falcons in the Arabian Gulf countries.

Annual scientific production from 1984 to 2024 showed an initially sparse research landscape, with 0–4 publications annually between 1984 and 1997, reflecting the nascent stage of falcon research in the region. This early phase was characterized by sporadic outputs, with some years producing no publications. From 1997 onward, a gradual increase was observed, with notable peaks occurring between late 1997 and early 2019, reaching up to seven publications annually. A more pronounced rise occurred between 2020 and 2024, culminating in a peak of nine publications in 2022 (Figure 3).

### Corresponding authors’ countries

**Figure 4:**
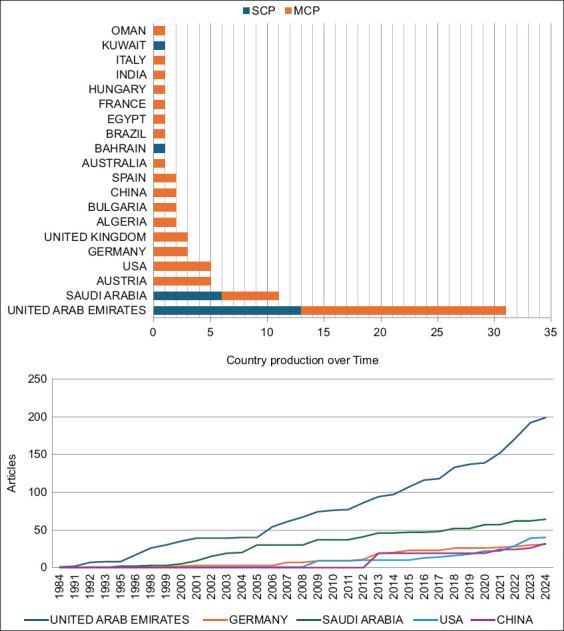
Corresponding author countries in falcon research in the Arabian Gulf Region (1984–2024). The bar graph illustrates the distribution of falcon research publications by the corresponding author’s country, highlighting the level of collaboration as either single-country publications (SCP) or multiple-country publications (MCP).

Analysis of corresponding authors’ countries demonstrated a predominant contribution from the UAE and Saudi Arabia. The UAE led in terms of corresponding author outputs, exhibiting strong participation in both single-country publications and multiple-country publications, highlighting its central role in promoting international collaborations (Figure 4). Saudi Arabia followed closely, also engaging extensively in international collaborations. Other significant contributors included Austria, the United States, and Germany, underscoring the global interest and collaborative nature of falcon research. Despite these efforts, the overall volume of research remains limited relative to the cultural significance of falcons in the region. The lower panel of Figure 4 illustrates the steady increase in research outputs from both the UAE and Saudi Arabia over time, reflecting their sustained investment in falconry and falcon research.

### Most relevant affiliations

**Figure 5:**
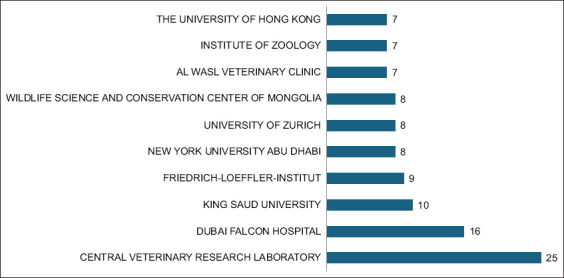
The most relevant affiliations in falcon research in the Arabian Gulf Region (1984-2024). The bubble chart illustrates the affiliations of corresponding authors who have significantly contributed to falcon research in the Arabian Gulf region.

Figure 5 highlights the most prominent institu-tional affiliations contributing to falcon research. Leading institutions included the Central Veterinary Research Laboratory, the Dubai Falcon Hospital, and King Saud University. Other notable contributors were the Friedrich-Loeffler Institute, New York University Abu Dhabi, and the University of Zurich, demonstrating substantial international institutional engagement.

### Most relevant authors

**Figure 6:**
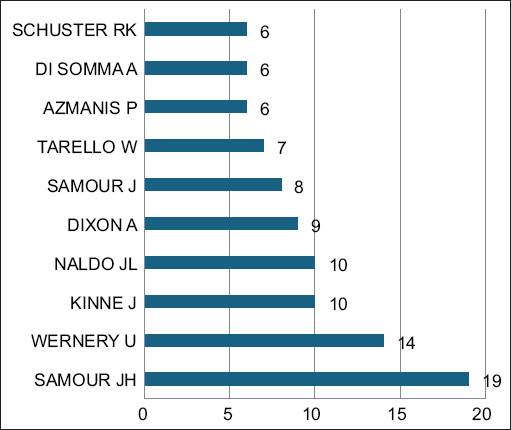
The most relevant authors in falcon research in the Arabian Gulf Region (1984–2024). The bubble chart represents the most prolific authors in the field of falcon research within the region, ranked by the number of documents they have author

As shown in Figure 6, Samour *et al*. [12] emerged as the most prolific author with 19 publications, primarily focusing on infectious diseases in falcons, including trichomoniasis. Dr. U. Wernery followed with 14 publications emphasizing the diagnosis of infectious diseases, notably avian paramyxovirus infections. Other leading contributors included Dr. J. Kinne and Dr. J.L. Naldo, each with 10 publications, and Dr. A. Dixon with nine publications.

### Most relevant sources

**Figure 7:**
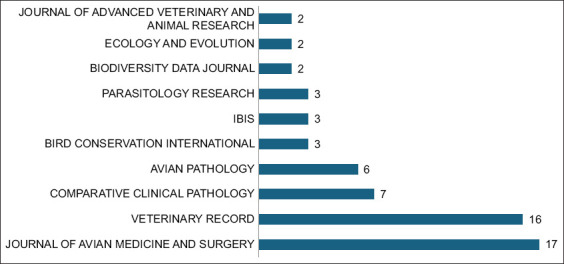
Most relevant sources of falcon research in the Arabian Gulf Region (1984–2024). The bubble chart shows the leading journals in which falcon research from the region has been published.

The Journal of Avian Medicine and Surgery emerged as the leading publication outlet with 17 articles (Figure 7), followed closely by the Veterinary Record with 16 articles. Other significant journals included Comparative Clinical Pathology (7 articles) and Avian Pathology (6 articles). Additional contributions from journals such as Bird Conservation International, Ibis, and Parasitology Research, though fewer in number, reflect the field’s interdisciplinary nature, encompassing veterinary medicine, pathology, conservation, and avian biology.

### Three-field plot analysis

**Figure 8:**
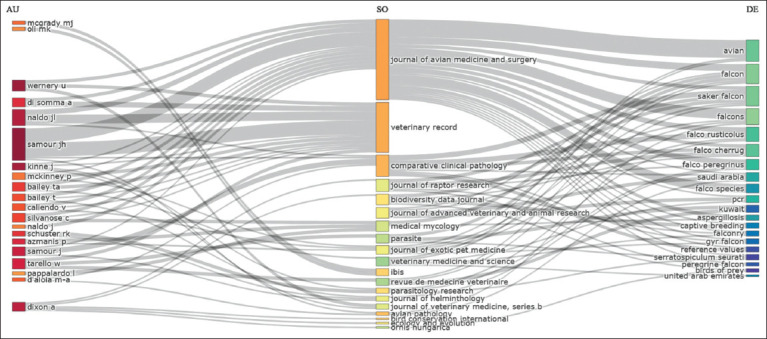
Three-field plot of Falcon research in the Arabian Gulf Region (1984–2024). This three-field plot illustrates the connections between the most prolific authors (AU), the journals in which they publish (SO), and the primary keywords (DE) associated with their research.

**Figure 9:**
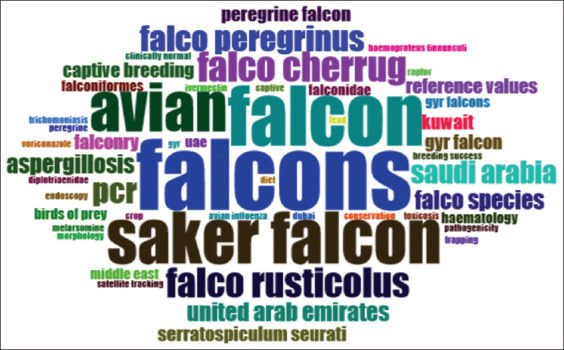
Word cloud of falcon research in the Arabian Gulf Region (1984–2024).

The three-field plot (Figure 8) illustrated the interconnected relationships among authors (AU), sources (SO), and keywords (DE) in falcon research. Prominent authors such as Dr. U. Wernery, Dr. J.H. Samour, and Dr. J.L. Naldo were closely associated with key journals, such as the Journal of Avian Medicine and Surgery and Veterinary Record. Frequently used keywords included “avian,” “falcon,” “Saker falcon,” “Falco rusticolus,” “Falco cherrug,” and “Falco peregrinus,” indicating a predominant focus on falcon health, biology, and conservation. These connections highlight a structured knowledge flow centered on veterinary and ecological aspects of falcon research. This observation was further corroborated by the word cloud analysis (Figure 9), where the size of each keyword reflected its frequency of occurrence.

### Trend topics

**Figure 10:**
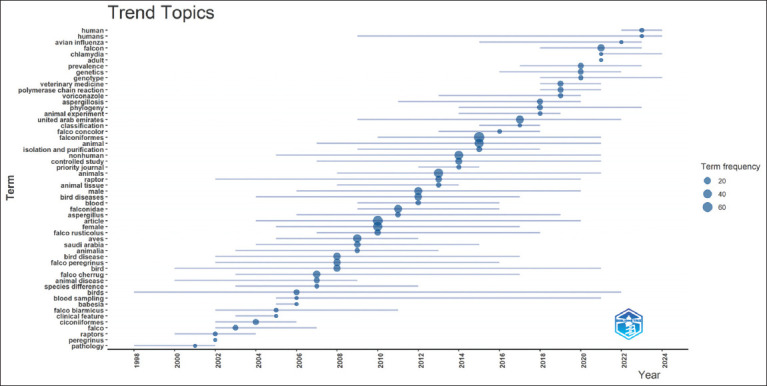
Trend topics in falcon research in the Arabian Gulf Region (1998–2024).

Trend topic analysis revealed significant thematic evolution over time (Figure 10). Early research predominantly addressed basic avian biology and clinical pathology, with keywords such as “raptors,” “species,” and “clinical features” dominating. The early 2010s marked a methodological shift with the introduction of molecular techniques, particularly polymerase chain reaction (PCR), fostering advances in genetic and disease diagnostic research. Since 2018, there has been an increased focus on epidemiology and infectious diseases, with key-words, such as “avian influenza,” “chlamydia,” and “aspergillosis” becoming prominent. The frequent emergence of location-specific terms such as “Saudi Arabia” and “United Arab Emirates” also underscores the regional relevance of the research.

### Thematic map

**Figure 11:**
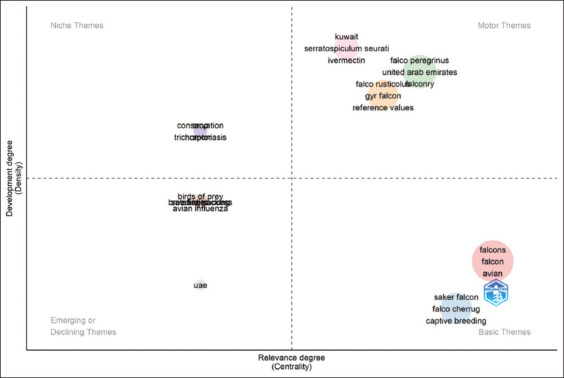
Thematic map of falcon research in the Arabian Gulf Region (1984–2024). The thematic map categorizes key research topics in falcon studies into four quadrants based on their development and relevance.

The thematic map (Figure 11) categorized research themes into four quadrants based on their development and relevance.

- Motor themes (well-developed and central) included specific falcon species, such as *Falco peregrinus* and *Falco rusticolus*, as well as practical topics, such as “falconry” and “reference values.”
- Niche themes such as “conservation” and “tricho-moniasis” were specialized but less integrated into broader research discussions.
- Basic themes (high relevance but underdeveloped) included “falcons,” “avian,” and “captive breeding,” highlighting critical areas needing further research investment.
- Emerging or declining themes included “avian influenza” and “birds of prey,” reflecting evolving research interests and priorities.

### International collaboration

**Figure 12:**
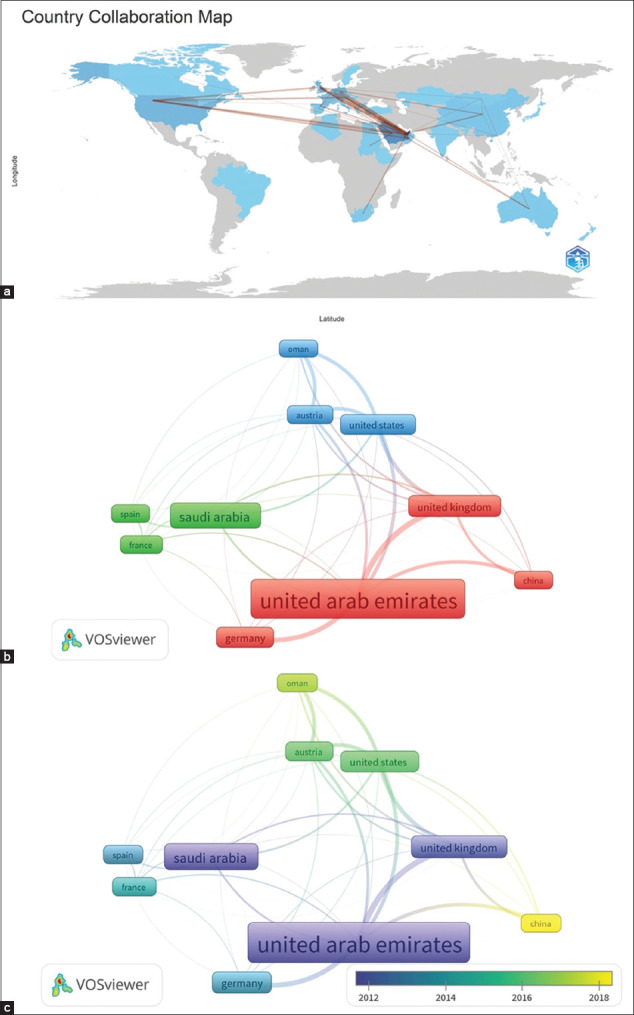
International collaboration in falcon research in the Arabian Gulf Region (1984–2024). The figure illustrates the patterns and evolution of international collaborations in falcon research related to the region. (a) The global map of collaborations, highlighting strong ties between the UAE and Saudi Arabia as well as key international partners. (b) A network visualization in which the UAE and Saudi Arabia are central nodes connected to other countries. (c) The time of evolution of these collaborations.

The global collaboration network (Figure 12a) revealed that the UAE and Saudi Arabia served as central hubs with extensive links to researchers in Europe (Germany, Austria, United Kingdom [UK]), North America (United States), and Asia (China). In Figure 12b, node size corresponded to the volume of collaborative outputs, with the UAE and Saudi Arabia appearing as the largest nodes. Bilateral collaborations with the USA, Germany, and the UK were particularly strong. Temporal analysis (Figure 12c) showed early collaborations with the UK, followed by partnerships with the USA, Austria, France, and Spain (green nodes), with more recent emerging collaborations involving China (yellow nodes), partially attributed to multinational affiliations and dual-country research efforts [19, 27].

### Keyword co-occurrence

**Figure 13:**
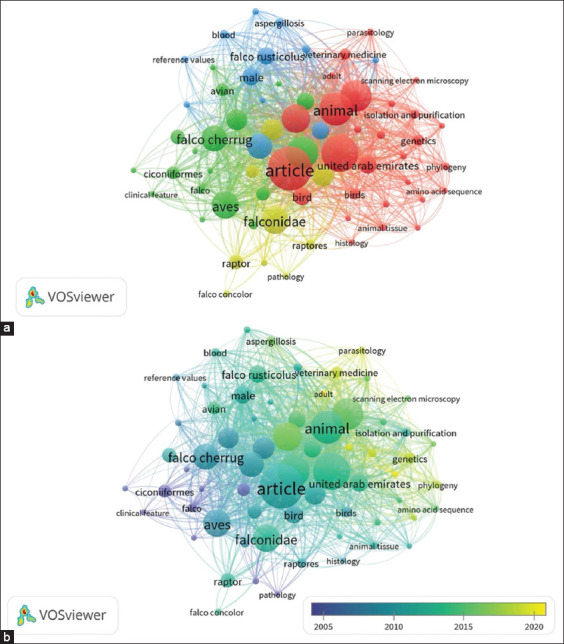
Keyword co-occurrence network in falcon research. (a) Network visualization of the co-occurrence of keywords in falcon research, grouped into distinct clusters. Each color represents a different cluster, indicating major thematic areas within the research field. (b) The time dimension to the co-occurrence network, with a color gradient indicating the average publication year of studies associated with each keyword. Keywords in blue represent research topics that have been established earlier (around 2005), while those in yellow indicate more recent research focus areas (around 2020).

The keyword co-occurrence network (Figure 13) classified research themes into four major clusters:

- Red cluster (Core biological and veterinary themes): Terms such as “animal,” “animals,” “falconiformes,” and “nonhuman,” reflecting biological and vete-rinary focuses.
- Green cluster (Falcon-specific and conservation themes): Species-specific research with keywords, such as “Falco peregrinus,” “Falco cherrug,” “saker falcon,” and “trichomoniasis.”
- Blue cluster (Diagnostics and laboratory analytical themes): Emphasis on pathology, microbiology, and diagnostic methods, including “aspergillosis,” “microbiology,” “blood,” “endoscopy,” and “refer-ence values.”
- Yellow cluster (Taxonomy, pathology, and morphological studies): Focus on taxonomy and morphology, with terms such as “raptor,” “falco-nidae,” “raptores,” and “ciconiiformes.”

The keyword network revealed that classical research areas, such as falcon biology, taxonomy, and veterinary medicine, have long been established, whereas newer areas are increasingly influenced by advancements in molecular biology, genetics, and human-falcon interaction studies. Emerging research topics highlighted the application of techniques such as scanning electron microscopy, phylogenetic analysis, and genetic diversity studies, indicating a shift toward more technology-driven research in falcon biology and conservation.

## DISCUSSION

This bibliometric analysis of falcon research in the Arabian Gulf countries reveals a steady yet limited growth in scholarly output over the past four decades, with the UAE and Saudi Arabia emerging as the primary contributors. These nations have demonstrated signif-icant collaborative research efforts, particularly in the areas of falcon health, conservation, and ecology. Despite aligning with the region’s cultural and ecolo-gical priorities, the modest volume of research relative to the iconic status of falcons suggests that the field remains underdeveloped. To strengthen falcon research, a comprehensive regional strategy is necessary, incorporating greater contributions from underrepresented Gulf countries such as Bahrain, Qatar, and Oman.

Thematically, the focus of falcon research has evolved from foundational aspects of biology and health toward more specialized topics, including molecular diagnostics and conservation strategies. Recent research trends highlight growing concerns over infectious diseases such as avian influenza, chlamydia, and aspergillosis, which pose significant threats to falcon populations. Furthermore, the shift toward molecular biology methodologies reflects a move toward more sophisticated approaches to understanding falcon genetics and disease susceptibility, potentially cont-ributing to improved conservation management. Emerging topics such as molecular diagnostics align with global priorities related to zoonotic disease surveillance and endangered species conservation. Nevertheless, critical gaps remain in fields such as captive breeding, genetics, and disease epidemiology, which are essential for ensuring sustainable falcon populations and effective health management.

Captive breeding programs are integral to the conservation of endangered falcon species, particularly in regions where wild populations are threatened by infectious disease outbreaks [7, 9, 18]. Advances in molecular diagnostics, including genome sequencing, read mapping, SNP calling, and filtering, provide powerful tools for assessing genetic variability and identifying potential risks [9, 18]. Moreover, these molecular tools enable early detection of infectious agents within breeding facilities, facilitating better health management and enhancing breeding success rates [7, 9, 28].

In this study, 126 documents were identified from the Arabian Gulf region, corresponding to an annual growth rate of 2.32%. However, at the global level, 1,074 falcon-related publications were retrieved from Scopus, with an annual growth rate of 2.01%. These findings indicate that the Arabian Gulf countries contributed approximately 11.73% to the global body of falcon research.

The analysis also revealed several persistent gaps that require attention to advance the field effectively. Despite the profound cultural and ecological significance of falcons in the Arabian Gulf, the overall research volume remains relatively low, indicating a limited breadth and depth of scientific inquiry. Key areas such as captive breeding, genetics, and epidemiology remain underexplored, despite their critical relevance. Furthermore, a regional bias is evident, with the majority of studies originating from the UAE and Saudi Arabia, while countries such as Bahrain, Kuwait, Qatar, and Oman remain underrepresented. This imbalance may hinder a comprehensive understanding of falcon conservation challenges and opportunities across the entire Gulf region. The disparity could be attributed to differences in research funding levels and cultural investment in falconry traditions. Although several falcon-related facilities exist in the lesser-represented countries, enhancing their research output may require strategic orientation and encouragement to actively disseminate their findings.

In terms of conservation policy, enhanced coop-eration among Gulf nations is essential to develop coherent, region-wide conservation plans that consider the transboundary nature of falcon migration and disease transmission. Such cooperation could involve the establishment of shared databases encompassing falcon genetics, disease surveillance outcomes, and complete genome sequences. In addition, the adoption of new technologies – including molecular diagnostics for health monitoring, genetic tools to guide captive breeding, and satellite telemetry for migration tracking – offers valuable opportunities for advancing evidence-based conservation strategies.

The trend topic analysis provides further insights into the shifting research landscape. In the early 2000s, research predominantly focused on basic avian biology and pathology, with keywords such as “avian,” “raptors,” “species,” “clinical features,” and “pathology” being most prominent [29, 30]. As research matured, more specialized topics, including the application of PCR for molecular studies, gained importance around 2012 [31, 32]. From 2018 onward, a marked increase in focus on diseases and epidemiology, particularly avian influenza, chlamydia, and aspergillosis, was obse-rved [28, 31, 33]. The frequent appearance of location-specific keywords such as “Saudi Arabia” and “United Arab Emirates” reflects the strong regional context of these research efforts.

The thematic map further elucidated the structure of falcon research:

- Motor themes (Upper right quadrant): Core, well-developed areas, such as *Falco peregrinus*, *Falco rusticolus*, “gyrfalcon,” “reference values,” “ivermectin,” and “The United Arab Emir-ates” [2, 7, 15, 34, 35], indicating practical and conservation-oriented research priorities.
- Niche themes (Upper left quadrant): Topics, such as “conservation” and “trichomoniasis” [5, 6, 12, 36], which, although well-developed, are less integrated into the broader research landscape.
- Basic themes (Lower right quadrant): Fundamental but underdeveloped topics, such as “falcons,” “avian,” “saker falcon,” and “captive breeding,” which are central but require further scientific investment.
- Emerging or declining themes (Lower left quadrant): Topics, such as “birds of prey,” “bearded vulture,” “avian influenza,” and references to the “UAE,” indicating areas either gaining traction or losing prominence.

This bibliometric analysis offers significant strengths, providing a comprehensive overview of falcon research evolution in the Arabian Gulf over four decades. The study emphasizes the cultural and ecological importance of falcons, aligning ongoing research efforts with regional conservation priorities. Through rigorous bibliometric methodologies, including keyword co-occurrence analysis and thematic mapping, the study identifies key research trends, leading contributors, and critical knowledge gaps. Moreover, the findings highlight the increasing internationalization of falcon research, particularly collaborations with the USA, Germany, China, and the UK, reinforcing the region’s integral role in the global falcon research community.

Nevertheless, certain limitations must be acknowledged. The study was confined to articles indexed in Scopus and published in English, potentially introducing language and publication bias and excluding relevant research from non-English sources and regional periodicals. Despite the cultural prominence of falcons, the relatively modest volume of research output over the four-decade period suggests a notable underdevelopment of the field. Future bibliometric studies should aim to include Arabic-language sources, regional journals, and gray literature to provide a more holistic analysis. Collaboration with regional researchers and conservation organizations could facilitate access to technical reports and unpublished datasets. Furthermore, cross-disciplinary approaches involving ecologists, veterinarians, geneticists, and conservation biologists are essential to address the existing knowledge gaps, particularly in disease epidemiology, falcon genetics, and conservation science. Regional cooperation efforts could be strengthened by incorporating emerging technologies such as whole-genome sequencing and artificial intelligence-driven disease prediction models. These strategies present promising avenues for advancing falcon health monitoring, early disease detection, and evidence-based conservation initiatives across the Arabian Gulf.

## CONCLUSION

This bibliometric analysis comprehensively mapped four decades of falcon research in the Arabian Gulf, encompassing 126 publications with an observed annual growth rate of 2.32%. The UAE and Saudi Arabia emerged as the leading contributors, reflecting substantial investments in falcon health, ecology, and conservation research. The thematic evolution demonstrated a shift from foundational studies on avian biology and clinical pathology toward more specialized areas, including molecular diagnostics, genetic studies, and the management of emerging infectious diseases such as avian influenza, chlamydia, and aspergillosis. Despite these advancements, the overall research output remains modest relative to the species’ cultural significance in the region.

This study provides valuable guidance for policymakers, conservationists, and research institutions in the Arabian Gulf by identifying critical gaps in falcon research output, thematic evolution, and collaboration networks. The findings highlight the need for greater investment in underrepresented research areas, such as captive breeding, genetics, and disease epidemiology, and emphasize the importance of integrating molecular diagnostics and emerging technologies into conservation strategies. Furthermore, the study supports the establishment of coordinated regional databases and enhanced cross-border collaborations to facilitate evidence-based conservation planning and disease surveillance. These insights have direct implications for strengthening falcon health management, biodiversity preservation, and the sustainable advancement of falconry heritage in the region.

A major strength of this study lies in its systematic and rigorous application of advanced bibliometric techniques, including keyword co-occurrence analysis, thematic mapping, and collaboration network visualization. These methods enabled a comprehensive overview of publication trends, the identification of influential authors, institutions, and journals, and the mapping of thematic developments and emerging research priorities. Furthermore, the analysis highlighted the increasing internationalization of falcon research, underscoring the region’s growing integration into global conservation and veterinary science initiatives.

Nonetheless, this study has certain limitations. The analysis was confined to publications indexed in the Scopus database and limited to English language articles, which may have introduced selection and language biases. Consequently, relevant regional research published in Arabic or in non-indexed journals may have been overlooked. In addition, the disproportionate representation of studies from the UAE and Saudi Arabia highlights a regional imbalance, with comparatively limited contributions from Bahrain, Kuwait, Qatar, and Oman, potentially affecting the generalizability of the findings across the entire Gulf region.

Future research efforts should aim to address these limitations by incorporating regional and non-English language publications, collaborating more extensively with local research centers, and encouraging the publication of technical reports and gray literature. Furthermore, a cross-disciplinary approach involving ecologists, veterinarians, geneticists, and conservation biologists is critical to closing the identified gaps, particularly in captive breeding, genetic diversity, and disease epidemiology. The adoption of emerging technologies such as whole-genome sequencing, artificial intelligence-based disease surveillance, and satellite telemetry for migration tracking offers promising pathways to enhance evidence-based conservation strategies. Strengthened regional cooperation will be pivotal in achieving coherent conservation policies that recognize the transboundary nature of falcon populations and emerging health threats in the Arabian Gulf.

## DATA AVAILABILITY

The supplementary data can be made available from the corresponding author upon request.

## AUTHORS’ CONTRIBUTIONS

MK and MA: Conceptualization and writing–original draft. AA and MK: Methodology. MK: Software. AA, MK, and MA: Formal analysis, data curation, and Writing–review and editing. All authors have read and approved the final manuscript.
